# Variants in *CDA* and *ABCB1* are predictors of capecitabine-related adverse reactions in colorectal cancer

**DOI:** 10.18632/oncotarget.3289

**Published:** 2015-01-21

**Authors:** Xandra García-González, Lucía Cortejoso, María I. García, Pilar García-Alfonso, Luis Robles, Cristina Grávalos, Eva González-Haba, Pellicer Marta, María Sanjurjo, Luis A. López-Fernández

**Affiliations:** ^1^ Department of Pharmacy, Hospital General Universitario Gregorio Marañón, Instituto de Investigación Sanitaria Gregorio Marañón, Madrid, Spain; ^2^ Department of Medical Oncology, Hospital General Universitario Gregorio Marañón, Instituto de Investigación Sanitaria Gregorio Marañón, Madrid, Spain; ^3^ Department of Medical Oncology, Hospital Universitario Doce de Octubre, Instituto de Investigación Sanitaria Doce de Octubre, Madrid, Spain

**Keywords:** Flouropyrimidine, toxicity, chemotherapy, genetics

## Abstract

Adverse reactions to capecitabine-based chemotherapy limit full administration of cytotoxic agents. Likewise, genetic variations associated with capecitabine-related adverse reactions are associated with controversial results and a low predictive value. Thus, more evidence on the role of these variations is needed. We evaluated the association between nine polymorphisms in *MTHFR*, *CDA*, *TYMS*, *ABCB1*, and *ENOSF1* and adverse reactions, dose reductions, treatment delays, and overall toxicity in 239 colorectal cancer patients treated with capecitabine-based regimens. The ABCB1*1 haplotype was associated with a high risk of delay in administration or reduction in the dose of capecitabine, diarrhea, and overall toxicity. *CDA* rs2072671 A was associated with a high risk of overall toxicity. *TYMS* rs45445694 was associated with a high risk of delay in administration or reduction in the dose of capecitabine, HFS >1 and HFS >2. Finally, *ENOSF1* rs2612091 was associated with HFS >1, but was a poorer predictor than *TYMS* rs45445694. A score based on *ABCB1-CDA* polymorphisms efficiently predicts patients at high risk of severe overall toxicity (PPV, 54%; sensitivity, 43%) in colorectal cancer patients treated with regimens containing capecitabine. Polymorphisms in *ABCB1*, *CDA*, *ENOSF1*,and *TYMS* could help to predict specific and overall severe adverse reactions to capecitabine.

## INTRODUCTION

Capecitabine is an oral fluoropyrimidine that delivers 5-fluorouracil (5-FU) to the tumor [1]. Both alone and in combination with other chemotherapeutic and biological agents, capecitabine is increasingly used in adjuvant and metastatic settings because it is easier to administer and has a more favorable toxicity profile than 5-FU [1]. Since their discovery in 1957, fluoropyrimidines have been the mainstay of treatment of colorectal cancer (CRC), a major cause of morbidity in developed countries [2]. 5-FU acts by inhibiting thymidylate synthase (TYMS) and incorporating drug metabolites into DNA and RNA, thus blocking DNA synthesis [3].

Capecitabine-treated patients commonly experience severe, even fatal, adverse drug reactions (ADRs) at some point during their treatment. These reactions often lead to dose reductions, delays in administration, and discontinuation of treatment [4, 5]. Although capecitabine is a prodrug of 5-FU, its toxicity profile is significantly different. While hematologic toxicity is most often associated with 5-FU, side effects such as diarrhea, nausea, vomiting, and hand-foot syndrome (HFS) are more commonly associated with capecitabine [6].

The toxicity of chemotherapeutic drugs is affected by factors such as age, performance status, organ dysfunction, and the presence of other co-morbidities. Interindividual genetic variability can also play an important role [7]. Many genes, nucleotides, antigens, and enzymes are known to be involved in the metabolism and efficacy of the drugs used in treatment of CRC. In addition, single-nucleotide polymorphisms (SNPs) lead to various outcomes in clinical practice [8]. Pharmacogenetics evaluates the effect of genetic variations on the individual response to and tolerability of therapy. Predicting the individual risk of toxicity for a particular drug could improve the quality of care. High-risk patients could be candidates for lower doses or alternative drugs in order to avoid toxicity [7]. Numerous gene polymorphisms have been associated with capecitabine-induced toxicity. For instance, *DPYD* variants have been extensively studied, and dosing guidelines have been suggested [9, 10] (http://www.pharmgkb.org/gene/PA166109594). However, the low frequency of toxicity-related alleles and the relatively frequent occurrence of severe ADRs to capecitabine indicate that other factors are involved in the risk of ADR. Laboratory tests have been designed for commercial or research purposes to predict the risk of fluoropyrimidine-induced toxicity. Nonetheless, they have all proven to be insufficiently accurate, thus stressing the need for new markers [11].

Various polymorphisms in *CDA*, *ABCB1*, *MTHFR*, and *TYMS* have been associated with capecitabine-induced ADRs, although findings are controversial and the evidence poor [7, 11-22]. The relationship between some of these genes and the development of toxicity to capecitabine is not clear. For instance, a meta-analysis describing an *ENOSF1* SNP in linkage disequilibrium with *TYMS* variants identified *ENOSF1* as a putative causal genetic variant for capecitabine-related toxicity [11]. However, the authors suggest that this finding needs to be confirmed in new cohorts.

We performed a prospective/retrospective study of a cohort of CRC patients treated with capecitabine-containing regimens in order to evaluate possible associations between severe ADRs to capecitabine and genomic variations in *CDA, ABCB1, ENOSF1*, *TYMS*, and *MTHFR*.

## RESULTS

A total of 239 capecitabine-treated patients were selected for the study. The baseline characteristics of the study population are shown in Table 1. The median age at diagnosis was 67 years (range, 30 to 88 years). Sex distribution was nearly homogeneous (54% men and 46% women). Patients had predominantly colon carcinoma (71.1%) and a good performance status (0-2, 98.7%), and 127 patients (53.1%) had metastatic disease. Combination regimens were more frequent than capecitabine monotherapy (74.5 vs 25.5%). Over half of the patients received oxaliplatin as part of a combination regimen (60.3%). Other concomitantly administered drugs included irinotecan and monoclonal antibodies (bevacizumab, cetuximab, and panitumumab).

Delay in administration, dose reduction, or withdrawal of the drug due to toxicity was common (70.7%) (Table 1). In clinical practice, a moderate HFS equal to 1 is often followed by a change in the treatment settings; therefore, we evaluated moderate-severe HFS (grade >1, 22.6%) and severe HFS (grade >2, 6.3%). Other frequently observed ADRs included severe diarrhea (grade >2, 10.9%) and severe hematological toxicity (grade >2, 7.1%).

Nine polymorphisms in five genes were genotyped for the 239 patients who fulfilled the inclusion criteria. No significant deviations from Hardy-Weinberg Equilibrium were detected, except for the SNP rs1045642 in *ABCB1* (*P*=0.01). Therefore, analyses of *ABCB1* were performed using haplotype *1 (rs1128503 C, rs2032582 G, and rs1045642 C). Univariate analysis revealed significant associations between multiple severe ADRs and the polymorphisms *CDA* rs2072671, *TYMs* rs45445694 and rs34489327, *ENOSF1* rs2612091, and ABCB1*1 (Table 2). *MTHFR* rs1801133 and rs1801131 showed no significant associations in this preliminary analysis and were therefore ruled out for subsequent testing. This analysis enabled us to identify putative risk alleles or variants.

**Table 1:** Patient characteristics

Characteristic	N (percentage)
Age	
Median age at diagnosis (range)	67 (30-88)
Sex	
Male	129 (54%)
Female	110 (46%)
Hospital	
H. Doce de Octubre	99(41.4%)
H. Gregorio Marañón	140 (58.6%)
Performance status	
≤2	236 (98.7%)
>2	2 (1.3%)
Tumor stage	
I-II	30 (12.6%)
III	82 (34.3%)
IV	127 (53.1%)
Type of cancer	
Colon	170 (71.1%)
Rectum	69 (28.9%)
Treatment setting	
Adjuvant	112 (46.8%)
Metastatic	127 (53.1%)
Number of cycles	
Median (range)	8 (1-58)
Regimen	
Monotherapy	61 (25.5%)
Combination	178 (74.5%)
Concomitant drug	
Oxaliplatin	144 (60.3%)
Irinotecan	28 (11.7%)
Antibodies	53 (22.1%)
Adverse reactions*	
Reduction/Delay/withdrawal treatment	169 (70.7%)
Nausea/Vomiting > 2	9 (3.8%)
Diarrhea > 2	26 (10.9%)
Hand-foot syndrome >2	15 (6.3%)
Hand-foot syndrome >1	54 (22.6%)
Hematological toxicity > 2	17 (7.1%)
Mucositis > 2	4 (1.7%)
Anorexia > 2	4 (1.7%)

* Adverse reaction graded according to National Cancer Institute Common Terminology Criteria for Adverse Events v4.0.

**Table 2:** Univariate comparisons between polymorphisms and adverse reactions to capecitabine

			Dose delay/reduction/withdrawal		Nausea/vomiting >2		HFS >1		HFS>2		Diarrhea >2		Hematological toxicity >2		Asthenia >2		Overall toxicity	
Gene	SNP	Genotype (n)	%	*P*	%	*P*	%	*P*	%	*P*	%	*P*	%	*P*	%	*P*	%	*P*
*CDA*	rs2072671	AA (96)	72.9	0.314	6.3	0.090	27.1	0.326	10.4	0.130	14.6	0.056	8.3	0.596	7.3	0.316	47.9	0.008**
		AC (106)	71.7		2.8		18.9		2.8		10.4		4.7		4.7		34.9	
		CC (37)	62.2		0.0		21.6		5.4		2.7		2.7		2.7		24.3	
*TYMS*
	rs45445694	2R/2R (44)	86.4	0.034*	0.0	0.477	45.5	0.001**	13.6	0.130	6.8	0.770	9.1	0.721	9.1	0.311	38.6	1.000
		2R/3R (116)	68.1		5.2		18.1		4.3		12.9		6.9		5.2		38.8	
		3R/3R (79)	65.8		3.8		16.5		5.1		10.1		6.3		3.8		38.0	
	rs34489327	del/del (20)	70.0	0.177	15.0	0.030*	5.0	0.011*	0.0	0.003**	20.0	0.512	0.0	0.428	0.0	0.022*	35.0	0.602
		del/ins (112)	65.2		3.6		17.9		5.4		9.8		11.6		2.7		37.5	
		ins/ins (107)	76.6		1.9		30.8		8.4		10.3		3.7		9.3		40.02	
*MTHFR*
	rs1801133	CC (100)	72.0	0.752	4.0	1.000	20.0	0.493	6.0	1.000	13.0	0.357	7.0	0.576	7.0	0.406	42.0	0.491
		CT (110)	70.0		3.6		24.5		6.4		10.0		9.1		4.5		45.5	
		TT (29)	69.0		3.4		24.1		6.9		6.9		0.0		3.4		37.9	
	rs1801131	AA (119)	72.3	0.821	1.7	0.169	21.8	0.805	5.0	0.669	7.6	0.184	7.6	1.000	4.2	0.821	36.1	0.523
		AC(103)	66.0		5.8		23.3		7.8		14.6		5.8		7.8		40.8	
		CC (17)	88.2		5.9		23.5		5.9		11.8		11.8		0.0		41.2	
*ENOSF1*	rs2612091	AA (76)	77.6	0.917	7.9	0.084	17.1	0.041*	6.6	0.437	13.2	0.367	6.6	1.000	1.3	0.020*	43.4	0.697
		AG (121)	62.0		1.7		21.5		4.1		10.7		8.3		5.8		39.9	
		GG (40)	82.5		2.5		35.0		12.5		7.5		5.0		12.5		42.5	
*ABCB1*	rs1128503 rs2032582 rs1045642	*1 (41)	90.2	0.002**	7.3	0.365	24.4	0.838	4.9	0.752	22.0	0.018*	12.2	0.181	9.8	0.246	65.9	<0.001***
		Other	66.7		3.0		22.2		6.6		8.6		6.1		4.5		32.8	

Given the inherent low statistical power of this kind of analysis and in order to obtain more robust significant associations, we performed a binary logistic regression analysis for those genotypes that initially rendered a statistically significant result. In the multivariate analysis, the *P* value was adjusted for sex, tumor stage, and hospital.

*CDA* rs2072671 was associated with overall toxicity (any ADR classed as grade 3 or higher). Homozygous AA patients presented a higher risk of overall toxicity than AC or CC patients (OR, 1.84; 95% CI, 1.06-3; P=0.029) (Table 3). A tendency toward HFS >2 was also observed for this SNP, although it was not statistically significant.

The *ABCB1* CGC haplotype for the SNPs rs1128503, rs2032582, and rs1045642 (ABCB1*1), respectively, was associated with the following delays in starting chemotherapy (any cycle), dose reduction, or withdrawal of capecitabine; severe diarrhea; and severe overall toxicity (Table 3). The association between ABCB1*1 and severe overall toxicity was particularly relevant (OR, 4.06; 95% CI, 1.97-8.38; *P*<0.001).

*TYMS* 2R (rs45445694) was significantly associated with moderate-severe HFS (grade >1) and severe HFS (grade >2) (Table 3). The *TYMS* 6ins allele was also associated with a higher risk of HFS >1 (*P*=0.011) and HFS >2 (*P*=0.003) in the univariate analysis (Table 2), although these associations disappeared after adjusting for sex, hospital, and type of cancer (Table 3).

Homozygous AA individuals harboring *ENOSF1* rs2612091 showed a higher risk of HFS >1 in the univariate analysis (*P*=0.041). This association remained statistically significant in the adjusted analysis (OR, 2.28; 95% CI, 1.10-4.76; *P*=0.027).

Given that *CDA* rs2072671 and *ABCB1* haplotypes (rs2072671, rs1128503, rs2032582, and rs1045642) were both significantly associated with severe overall toxicity, a *CDA-ABCB1* score was calculated based on the number of risk alleles (from 0 to 8). A *CDA-ABCB1* score >5 predicted overall toxicity with a positive predictive value of 54.05%, negative predictive value of 68.48%, sensitivity of 43.47%, and specificity of 76.87%.

**Table 3:** Analysis by logistic regression of previous significant associations

	OR	CI	*Padj*
*ABCB1* (Ref: no ABCB1*1)			
Delay/reduction/withdrawal	4.49	1.53-13.19	0.006**
Diarrhea >2	3.16	1.28-7.79	0.012*
Hand-foot syndrome >1	1.11	0.50-2.46	0.798
Hand-foot syndrome >2	0.72	0.15-3.37	0.673
Hematological toxicity >2	2.16	0.71-6.56	0.173
Asthenia >2	2.46	0.69-8.80	0.165
Overall toxicity	4.06	1.97-8.38	<0.001***
*CDA* rs2072671 (Ref: AC/CC)			
Delay/reduction/withdrawal	1.25	0.69-2.25	0.460
Diarrhea >2	1.83	0.79-4.24	0.157
Hand-foot syndrome >1	1.56	0.83-2.94	0.163
Hand-foot syndrome >2	2.89	0.93-8.98	0.066
Hematological toxicity >2	1.38	0.50-3.80	0.531
Asthenia >2	1.40	0.44-4.49	0.566
Overall toxicity	1.84	1.06-3.18	0.029*
*TYMS* rs45445694 (Ref: 2R-3R /3R-3R)			
Delay/reduction/withdrawal	3.07	1.23-7.70	0.016*
Diarrhea >2	0.54	1.15-1.90	0.336
Hand-foot syndrome >1	3.78	1.86-7.76	<0.001***
Hand-foot syndrome >2	3.63	1.18-11.22	0.025*
Hematological toxicity >2	1.40	0.43-4.56	0.576
Asthenia >2	2.14	0.60-7-60	0.341
Overall toxicity	0.97	0.49-1.93	0.937
*TYMS* rs34489327 (Ref: del-ins/ins-ins)			
Delay/reduction/withdrawal	0.99	0.36-2.71	0.981
Diarrhea >2	2.24	0.68-7.37	0.186
Hand-foot syndrome >1	0.16	0.02.1.23	0.078
Hand-foot syndrome >2	0.58	0.27-NA	0.628
Hematological toxicity >2	0.43	0.37-NA	0.429
Asthenia >2	2.13	0.60-7.60	0.241
Overall toxicity	0.92	0.35-2.42	0.862
ENOSF1 rs2612091 (Ref: GA/AA)			
Delay/Reduction/withdrawal	2.21	0.92-5.27	0.074
Diarrhea >2	0.60	0.17-2.12	0.431
Hand-foot syndrome >1	2.28	1.10-4.76	0.027*
Hand-foot syndrome >2	2.53	0.80-8.02	0.114
Hematological toxicity >2	0.62	0.14-2.84	0.541
Asthenia >2	3.15	0.94-10.57	0.063
Overall toxicity	0.91	0.45-1.82	0.789

Adjusted (*Padj*) P values were calculated using binary logistic regression;

* *P* < 0.05;

** *P* < 0.01;

*** *P* <0.001; Ref: Reference.

## DISCUSSION

Cancer cells are the target of chemotherapy. Adverse effects to cancer therapy result from damage to healthy cells. Selective targeting of cancer cells has been investigated to decrease the side effects of chemotherapy [23, 24]. However, to date, no valid approaches have been implemented in clinical practice. Identification and validation of genetic biomarkers with the aim of preventing severe ADRs to chemotherapeutic drugs could prove crucial in helping oncologists to select the best treatment for their patients. Since capecitabine is one of the most commonly used drugs in the treatment of CRC, a specific tool that would make it possible to predict the risk of toxicity for individual patients would be very useful. All the polymorphisms analyzed in this study have been associated with toxicity in the literature. However, current evidence is insufficient to apply those results in clinical practice. In this study, we demonstrated that functional genetic variants in *TYMS*, *ENOSF1*, and *ABCB1* were associated with severe toxicity.

The genetic polymorphisms rs34489327 and rs45445694 in *TYMS* have been studied extensively to determine the patient's response to fluoropyrimidine-based chemotherapy, although fewer studies have focused on their association with toxicity. In this cohort of capecitabine treated CRC patients we found a correlation between the *TYMS* polymorphism rs45445694 and HFS (grade >1 and grade >2). This result confirms previous findings, thus suggesting a clear association that could increase the level of evidence for using these biomarkers in clinical practice [11, 25-27].

*ENOSF1* was recently proposed as a regulator of *TYMS* expression via antisense mechanisms because the sequences of both genes are complementary [11]. In addition, an association was found between *ENOSF1* rs2612091 and capecitabine-related severe toxicity, mainly HFS. Therefore, a more relevant role has been suggested for *ENOSF1*—as opposed to *TYMS*—in capecitabine-related toxicity. We observed an increased risk of HFS grade >1 in patients carrying the G variant in homozygosis in *ENOSF1* rs2612091. However, this association did not remain statistically significant when HFS grade >2 was analyzed. The results in our cohort suggest that *TYMS* rs45445694 (OR, 3.63) is a better predictor of severe HFS than *ENOSF1* rs2612091 (OR, 2.53).

We did not find any significant associations between capecitabine-related toxicity and the *MTHFR* polymorphisms 677C>T and 1298A>C. It has been suggested that the effects of *MTHFR* are masked in cases of high serum levels of active folate [28]. Although capecitabine is not combined with leucovorin, greater folate intake has been related to a Mediterranean diet, and higher folate levels may explain the lack of effect of the *MTHFR* polymorphisms 677C>T and 1298A>C [29].

We also found that *CDA* 79A>C was linked to overall toxicity and observed a trend toward severe diarrhea. *CDA* plays an important role in the conversion of capecitabine to 5-FU, and variants in this gene have been related to capecitabine-induced toxicity [11, 22, 30]. *CDA* 79A>C has been associated with *CDA* activity and with toxicity to cytarabine, which is also metabolized by *CDA* [31, 32]. In contrast to our findings, those of other studies did not show a connection between this SNP and toxicity induced by capecitabine [11, 22] and fluoropyrimidines [33]. The variables considered responsible for these discrepant results include differences in study design, sample size, criteria for establishing a cut-off for severe ADRs, as well as variations in concomitant medication [34].

ABCB1 is an ATP-dependent drug efflux transporter for a huge variety of substrates [35]. Although capecitabine has not been clearly identified as one of them, *ABCB1* expression has been associated with resistance to 5-FU in modified cell lines [36]. The authors showed associations between the haplotype ABCB1*1 and an increased probability of reducing the dose of capecitabine, delaying initiation of therapy, or withdrawing the drug altogether, as well as of diarrhea and overall toxicity. Nevertheless, we were not able to reproduce the association with HFS previously obtained in a smaller cohort [20].

A score covering several polymorphisms located in at least two genes has been designed to predict adverse reactions to fluoropyrimidines. Afzal *et al* used polymorphisms in *TYMS* and *MTHFR* to predict gastrointestinal toxicity in 5-FU-treated patients [19], while Rosmarin *et al* developed a test to predict overall toxicity with polymorphisms in *TYMS* and *DPYD* in capecitabine-treated patients [11]. Jennings *et al* suggested a predictive test for early adverse events by analyzing *TYMP* and *DPYD* variants as a signature (sensitivity of 45.5% and a positive predictive value of 39.9%) [33]. Our results for predicting overall toxicity using the *CDA-ABCB1* score are similar to those obtained in the studies, although they do not take into consideration the main factor contributing to toxicity in fluoropyrimidine-based treatments, namely, *DPYD* variants. All other authors have included *DPYD* variants in their scores. In our preliminary analyses, we genotyped three SNPs in *DPYD*, namely, rs67376798, rs55886062, and rs3918290. However, we found only two patients who were heterozygous for rs67376798, and they did not experience any relevant toxicity. Given the low frequency of *DPYD* variants and our limited sample size, we decided not to include *DPYD* variants in our score. We suggest that using *CDA* and *ABCB1* polymorphisms could improve the performance of existing predictors for capecitabine-induced toxicity. Alternatively, the novel strategy of using gene-specific scores for different ADRs could help to improve the prediction of a specific ADR. Identifying patients with a very high risk for developing ADRs could be useful when investigating new strategies, such as decreasing chemotherapy dosage in order to reduce toxicity without decreasing efficacy.

In summary, polymorphisms in *TYMS*, *CDA*, *ENOSF1*, and *ABCB1* are associated with ADRs to capecitabine-based chemotherapy in CRC patients. Polymorphisms in *CDA* and *ABCB1* should be added to current models to predict overall toxicity. Therefore, genotyping of these variants could help with the implementation of pharmacogenetics in clinical practice.

## METHODS

### Patients

Samples from patients were provided by the oncology departments of two tertiary teaching hospitals (Hospital General Universitario Gregorio Marañón and Hospital Universitario Doce de Octubre) and by HGM BioBank, which is a member of ReTBioH. Samples were processed following current procedures and frozen immediately on reception. 

The inclusion criteria were diagnosis of CRC (any stage), a previous capecitabine-containing regimen in any line of treatment, and age over 18 years. The exclusion criteria were as follows: noncompliance with any of the inclusion criteria, kidney or liver damage prior to treatment, and less than 2 months of treatment, unless higher-grade severe ADRs occurred (grade ≥2 for HFS and grade≥3 for the rest).

The demographic and clinical data collected included sex, age, colon or rectal cancer, disease stage, World Health Organization Performance Status score, treatment setting (adjuvant or metastatic), and other drugs received as part of the chemotherapy regimen. Capecitabine-related adverse reactions including nausea and vomiting, diarrhea, mucositis, hematological toxicity, anorexia, asthenia, and HFS were recorded and graded according to the Common Terminology Criteria for Adverse Events v4.0 (CTCAE) of the National Cancer Institute. All patients were genotyped for polymorphisms in the following genes: *MTHFR*, *TYMS*, *CDA*, *ABCB1*, and *ENOSF1*. Associations between the polymorphisms and the ADRs were analyzed. This study was conducted in accordance with the Helsinki Declaration and was approved by the ethics committees of Hospital General Universitario Gregorio Marañón (CEIC-A1) and Hospital Universitario Doce de Octubre (CEIC-A11). Informed consent was obtained from all patients.

### DNA isolation

Genomic DNA was isolated from 200 μl of whole blood using the High Pure PCR template preparation kit (Roche Applied Sciences, Penzberg, Germany). The DNA concentration was measured using a NanoDrop spectrophotometer (ThermoScientific, Waltham, Massachusetts, USA).

### Genotype analysis

The polymorphisms rs1801133 (*MTHFR* C677T), rs1801131 (*MTHFR* A1298C), rs1128503 (*ABCB1* 1236C>T), rs2032582 (*ABCB1* 2677G>T/A), rs1045642 (*ABCB1* 3435C>T), and rs2072671 (*CDA* 79C/T) were genotyped using SNaPshot, as previously described [20]. The oligonucleotides used in the multiplex PCR reaction were as follows: rs1801133-F TCA CAA AGC GGA AGA AGT, rs1801133-R GCC TCT CCT GAC TGT ATC, rs1801131-F CTT TGT GAC CAT TCC GGT T, rs1801131-R TTT GGG GAG CTG AAG GAC TA, rs1128503-F CTC GAC TCA CCA CAC CAA TG, rs1128503-R TAT CCT GTG TCT GTG AAT TGC C, rs2032582-F TAG TTT GAC TCA CCT TCC CGG, rs2032582-R GGC TAT AGG TTC CAG GCT TG, rs1045642-F CAT GCT CCC AGG CTG TTT AT, rs1045642-R GTA ACT TGG CAG TTT CAG TG, rs2072671-F CTG AAG CCT GAG TGT GTC CA, and rs2072671-R CCA TCC AAC TTC CTT CCT CA. The oligonucleotides used in the SNaPshot reactions were as follows: rs1801133SNAP AGA ATG TGT CAG CCT CAA AGA AAA AGC TGC GTG ATG ATG AAA TCG, rs1801131SNAP TCC GGT TTG GTT CTC CCG AGA GGT AAA GAA CAA AGA CTT CAA AGA CAC TT, rs1128503SNAP GCC CAC TCT GCA CCT TCA GGT TCA G, rs2032582SNAP GAC AAG CAC TGA AAG ATA AGA AAG AAC TAG AAG GT, rs1045642SNAP TGA CTC GAT GAA GGC ATG TAT GTT GGC CTC CTT TGC TGC CCT CAC, and rs2072671SNAP TTT TTC CTG AGT GTG TCC AGC AGC TGC TGG TTT GCT CCA AGG AGG CCA AG.

The SNP rs34489327 (*TYMS* 6del) was genotyped using PCR amplification length polymorphism analysis with the primers 5′ 6-FAM- CTCAAATCTGAGGGAGCTGAG 3′ and 5′ GCAGAACACTTCTTTATTATAG 3′ and capillary electrophoresis. Genomic DNA (20 ng) was used as a PCR template under the following conditions: 95°C for 5 min, 35 cycles of 95°C for 30 s, 60°C for 40 s, 72°C for 90 s, and a final extension of 72°C for 5 min. PCR products were purified with ExoSapIt [20]. Purified PCR product (1 μL) was loaded onto an ABI Prism 3100 Genetic Analyzer and analyzed using PeakScan v1.0 (Life Technologies, Carlsbad, California, USA).

The SNP 45445694 in *TYMS* was analyzed by PCR amplification of the region containing it and analysis of the length of the amplification products. The oligonucleotides used in the PCR were 5′ GTGGCTCCTGCGTTTCCCCC 3′ and 5′ GCTCCGAGCCGGCCACAGGCA 3′. PCR parameters were the same as for *TYMS* 6del. The amplified product was purified using the High Pure PCR product purification kit and subsequently digested with the restriction enzyme HaeIII (both from Roche Applied Science, Penzberg, Germany). For this purpose, 8 μL of the amplification product was incubated with 1 μL of HaeIII and 1 μL of restriction buffer at 37°C for 1 h. After this period, the enzyme was inactivated by heating at 65°C for 15 min. Finally, the amplification products and the products resulting from the digestion were analyzed using the 2100 Bioanalyzer DNA1000 and the reagents kit (Agilent Technologies, Santa Clara, California, USA). Thus, the amplification of the region containing two repeats of 28 bp corresponds to a 214-bp band while 3 repeats would correspond to a 242-bp band.

*ENOSF1* rs2612091 was genotyped using a TaqMan probe in a StepOne Plus Real Time PCR system (Life Technologies, Carlsbad, California, USA). Allele discrimination was performed using StepOne software v2.3.

Hardy-Weinberg equilibrium was analyzed to detect deviations in genotype frequency [37].

### Statistical analysis

All data were analyzed using the Statistical Package for the Social Sciences v.15 (SPSS, Inc). A linear-by-linear association chi-squared test was used to study the univariate associations between polymorphisms and the grade of adverse events. A *P* value <0.05 was considered statistically significant. The odds ratio (OR) and 95% confidence intervals (CI) were reported in the multivariate logistic regression models for associations that were statistically significant in the univariate analysis. For *ABCB1* variants, we performed a haplotype-based test. The variables analyzed in the models included genotype, sex, tumor stage, and hospital.

Based on the results of the multivariate analysis, a score test was designed based on the number of risk alleles for overall toxicity in *ABCB1* and *CDA*. Negative and positive predictive values, specificity, and sensitivity were calculated.
